# Loss of DNA methylation is related to increased expression of miR-21 and miR-146b in papillary thyroid carcinoma

**DOI:** 10.1186/s13148-018-0579-8

**Published:** 2018-11-20

**Authors:** Isabella Maria Dias Payão Ortiz, Mateus Camargo Barros-Filho, Mariana Bisarro dos Reis, Caroline Moraes Beltrami, Fabio Albuquerque Marchi, Hellen Kuasne, Luísa Matos do Canto, Julia Bette Homem de Mello, Cecilie Abildgaard, Clóvis Antônio Lopes Pinto, Luiz Paulo Kowalski, Silvia Regina Rogatto

**Affiliations:** 1 International Research Center-CIPE, A.C. Camargo Cancer Center, Taguá Street 440, São Paulo, 01508-010 Brazil; 2 Department of Clinical Genetics, Vejle Hospital, Institute of Regional Health Research, University of Southern Denmark, Beriderbakken 4, 7100 Vejle, Denmark; 3 Department of Pathology, A.C. Camargo Cancer Center, Professor Antonio Prudente Street 211, São Paulo, 01509-900 Brazil; 4 Department of Head and Neck Surgery and Otorhinolaryngology, A.C. Camargo Cancer Center, Professor Antonio Prudente Street 211, São Paulo, 01509-900 Brazil

**Keywords:** DNA methylation, microRNA, miR-146b, miR-21, Papillary thyroid, Carcinoma

## Abstract

**Background:**

DNA methylation in miRNA genes has been reported as a mechanism that may cause dysregulation of mature miRNAs and consequently impact the gene expression. This mechanism is largely unstudied in papillary thyroid carcinomas (PTC).

**Methods:**

To identify differentially methylated miRNA-encoding genes, we performed global methylation analysis (Illumina 450 K), integrative analysis (TCGA database), data confirmation (pyrosequencing and RT-qPCR), and functional assays.

**Results:**

Methylation analysis revealed 27 differentially methylated miRNA genes. The integrative analyses pointed out miR-21 and miR-146b as potentially regulated by methylation (hypomethylation and increased expression). DNA methylation and expression patterns of miR-21 and miR-146b were confirmed as altered, as well as seven of 452 mRNAs targets were down-expressed. The combined methylation and expression levels of miR-21 and miR-146b showed potential to discriminate malignant from benign lesions (91–96% sensitivity and 96–97% specificity). An increased expression of miR-146b due to methylation loss was detected in the TPC1 cell line. The miRNA mimic transfection highlighted putative target mRNAs.

**Conclusions:**

The increased expression of miR-21 and miR-146b due to loss of DNA methylation in PTC resulted in the disruption of the transcription machinery and biological pathways. These miRNAs are potential diagnostic biomarkers, and these findings provide support for future development of targeted therapies.

**Electronic supplementary material:**

The online version of this article (10.1186/s13148-018-0579-8) contains supplementary material, which is available to authorized users.

## Background

Papillary thyroid carcinoma (PTC) is the most frequent thyroid malignant neoplasm and is responsible for the increased incidence of thyroid cancer worldwide [1, 2]. The main genetic alterations described in PTC are *BRAF* and *RAS* mutations and *RET* rearrangements [3]. Furthermore, *TERT* promoter mutations have been associated with more aggressive thyroid carcinomas [4, 5], especially in tumors harboring *BRAF* mutations [4].

DNA methylation and microRNA (miRNA) are events capable to regulate the expression of genes related to cancer development, as previously reported in PTC [6–9]. A recent study conducted by our group identified DNA methylation alterations related to prognosis in well differentiated thyroid lesions [10]. Using a robust methylation platform in 141 thyroid samples (non-neoplastic tissue, benign lesions, and carcinomas), we developed a prognostic classifier based on 21 CpGs. This classifier was able to distinguish well-differentiated thyroid carcinomas of patients showing worse prognosis (relapse during the follow-up) from those with good prognosis (without relapse in the follow-up) [10].

Similar to protein-encoding genes, miRNAs are transcribed and could be targets of epigenetic events that modulate their expression [11]. In the last few years, the number of miRNAs described as regulated by DNA methylation increased substantially [11–13]. However, the characterization of this epigenetic modification and its functional role in the control of miRNA expression are poorly explored in PTC. To our knowledge, only one study described miRNAs putatively regulated by methylation in thyroid cancer [7]. Nonetheless, neither confirmation nor functional experiments have been developed in this field. This knowledge can contribute to better understanding of PTC biology leading to the discovery of biomarkers and new therapeutic strategies.

Herein, a comprehensive DNA methylation data from PTC and matched NT (non-neoplastic tissue) samples, previously described by our group [8, 10], were re-evaluated focusing in the identification of miRNA genes potentially regulated by methylation. External molecular dataset from TCGA were assessed to perform integrative analysis using DNA methylation, miRNAs expression, and target mRNAs data. The miRNA-coding genes, *MIR21* and *MIR146B*, were further investigated in an independent sample set, and functional assays were carried out in PTC cell lines.

## Results

### MicroRNA genes differentially methylated in PTC

**Fig. 1:**
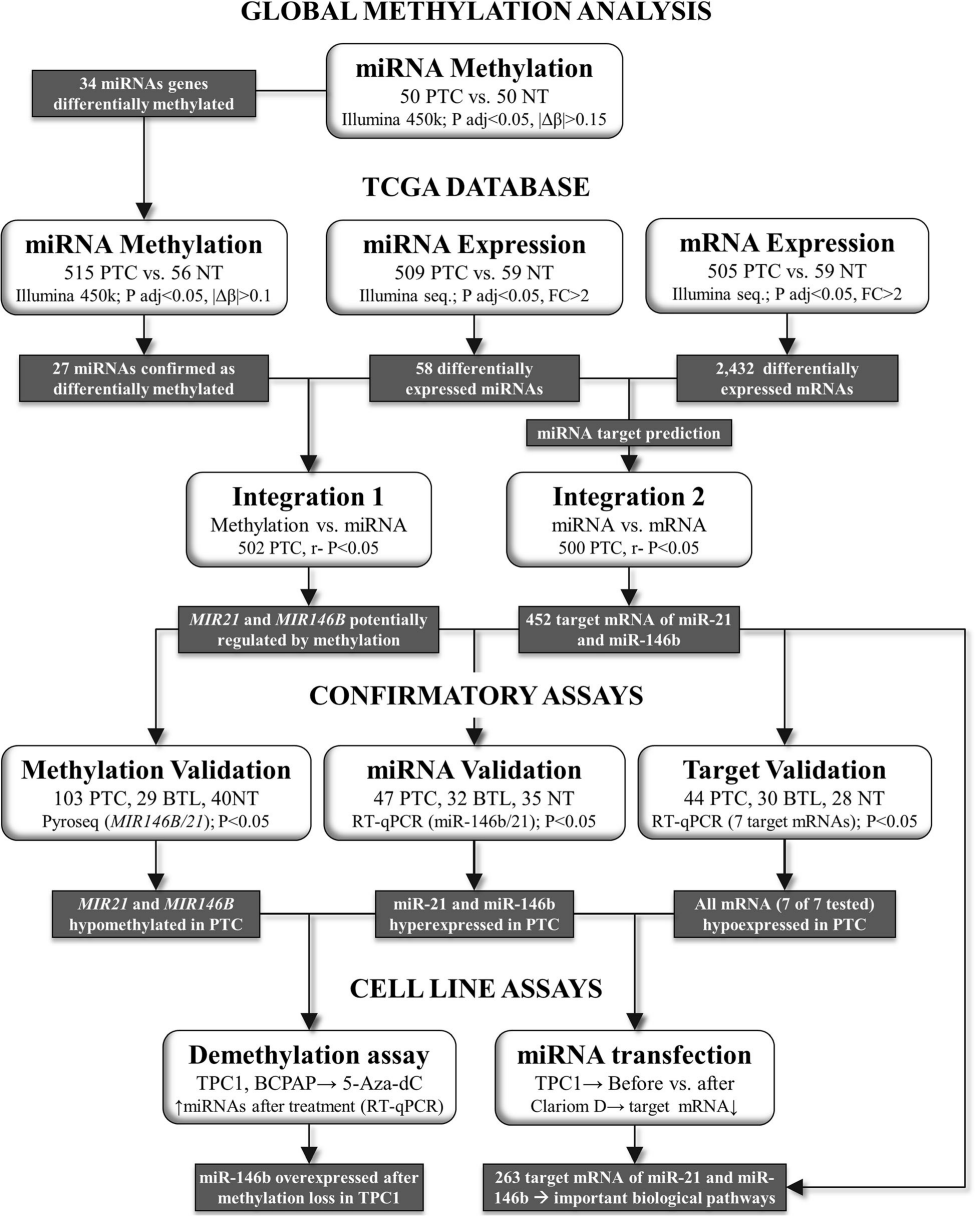
Flowchart showing the strategies and results obtained using bioinformatic tools to identify miRNA genes potentially regulated by methylation, their target mRNA and data confirmation studies. PTC, papillary thyroid carcinoma; NT, non-neoplastic thyroid tissue; BTL, benign thyroid lesion; *P* adj, adjusted *P* value, |Δβ|, delta beta; FC, fold-change; *r*−, negative correlation; *Pearson test

The main strategies to identify miRNAs potentially regulated by methylation are depicted in Fig. 1. DNA methylation analysis comparing paired PTC (*N* = 50) and NT (*N* = 50) samples revealed 50 CpG probes (34 miRNA genes) differentially methylated, of which 86% (42 probes mapped in 27 miRNA genes) was confirmed using the TCGA database (Table 1). A supervised hierarchical clustering analysis with all 42 probes revealed an enrichment of hypomethylation in both, our PTC cases and in the TCGA dataset (Additional file 1: Figure S1).

**Table 1:** CpG probes mapped in miRNA-coding genes differentially methylated in PTC compared to NT

Probe (ID)	miRNA gene	Genomic functional distribution	CpG context	Internal data			TCGA data		
				Delta β	*P*	*P* adj.	Delta β	*P*	FDR
cg19019198	*MIRLET7G*	TSS200		− 0.36	< 1e-07	< 1e-07	− 0.49	< 1e-07	< 1e-07
cg07181702	*MIR21*	Body		− 0.19	< 1e-07	< 1e-07	− 0.20	< 1e-07	< 1e-07
cg04276626	*MIR21*	TSS200		− 0.18	< 1e-07	< 1e-07	− 0.16	< 1e-07	< 1e-07
cg02515217	*MIR21*	TSS200		− 0.17	< 1e-07	< 1e-07	− 0.16	< 1e-07	< 1e-07
cg15759721	*MIR21*	Body		− 0.17	< 1e-07	< 1e-07	− 0.14	< 1e-07	< 1e-07
cg04805065	*MIR33B*	TSS1500	Shore	− 0.25	< 1e-07	< 1e-07	− 0.29	< 1e-07	< 1e-07
cg09186408	*MIR33B*	TSS1500	Shore	− 0.20	< 1e-07	< 1e-07	− 0.23	< 1e-07	< 1e-07
cg19619576	*MIR33B*	TSS1500	Shelf	− 0.16	< 1e-07	1e-07	− 0.21	< 1e-07	< 1e-07
cg01243312	*MIR128–1*	Body		0.20	< 1e-07	< 1e-07	0.23	< 1e-07	< 1e-07
cg10734581	*MIR134*	TSS1500		− 0.19	< 1e-07	< 1e-07	NA	NA	NA
cg15857661	*MIR146B*	TSS200	Shelf	− 0.33	< 1e-07	< 1e-07	− 0.34	< 1e-07	< 1e-07
cg05858126	*MIR146B*	TSS200	Shelf	− 0.32	< 1e-07	< 1e-07	− 0.33	< 1e-07	< 1e-07
cg05251190	*MIR146B*	TSS200	Shelf	− 0.30	< 1e-07	< 1e-07	− 0.29	< 1e-07	< 1e-07
cg13442016	*MIR146B*	Body	Shelf	− 0.29	< 1e-07	< 1e-07	− 0.29	< 1e-07	< 1e-07
cg09701700	*MIR146B*	TSS1500	Shelf	− 0.20	< 1e-07	< 1e-07	− 0.19	< 1e-07	< 1e-07
cg13309012	*MIR155*	Body		− 0.30	< 1e-07	< 1e-07	− 0.30	< 1e-07	< 1e-07
cg13449535	*MIR200B*	TSS1500	Shore	− 0.25	< 1e-07	< 1e-07	− 0.37	< 1e-07	< 1e-07
cg14144728	*MIR200B*	TSS1500	Shore	− 0.23	< 1e-07	< 1e-07	− 0.21	< 1e-07	< 1e-07
cg08096702	*MIR211*	TSS1500		− 0.29	< 1e-07	< 1e-07	− 0.34	< 1e-07	< 1e-07
cg11721554	*MIR377*	TSS1500	Shelf	− 0.17	< 1e-07	< 1e-07	− 0.20	< 1e-07	< 1e-07
cg05138957	*MIR377*	TSS1500	Shelf	− 0.15	< 1e-07	< 1e-07	− 0.19	< 1e-07	< 1e-07
cg21513316	*MIR410*	Body	Island	− 0.16	< 1e-07	< 1e-07	− 0.14	< 1e-07	< 1e-07
cg20547131	*MIR412*	TSS1500	Shore	− 0.17	< 1e-07	< 1e-07	− 0.15	< 1e-07	< 1e-07
cg14910227	*MIR495*	Body		− 0.19	< 1e-07	< 1e-07	− 0.27	< 1e-07	< 1e-07
cg11978784	*MIR512–1*	TSS1500		− 0.17	< 1e-07	< 1e-07	NA	NA	NA
cg10583119	*MIR518D*	TSS1500		− 0.18	< 1e-07	< 1e-07	− 0.22	< 1e-07	< 1e-07
cg17670263	*MIR520C*	TSS1500		− 0.21	< 1e-07	1e-07	− 0.27	< 1e-07	< 1e-07
cg05865548	*MIR543*	TSS200		− 0.16	< 1e-07	< 1e-07	− 0.22	< 1e-07	< 1e-07
cg04522625	*MIR548A2*	Body		0.24	< 1e-07	< 1e-07	0.30	< 1e-07	< 1e-07
cg18697991	*MIR548A2*	Body		0.19	< 1e-07	< 1e-07	0.20	< 1e-07	< 1e-07
cg03221073	*MIR548F1*	Body		− 0.41	< 1e-07	< 1e-07	− 0.34	< 1e-07	< 1e-07
cg25874148	*MIR548F5*	Body		− 0.28	< 1e-07	< 1e-07	NA	NA	NA
cg01719718	*MIR548F5*	Body		− 0.17	< 1e-07	2e-07	− 0.18	< 1e-07	< 1e-07
cg05878887	*MIR548G*	Body		− 0.31	< 1e-07	< 1e-07	− 0.33	< 1e-07	< 1e-07
cg21175685	*MIR548G*	Body		− 0.31	< 1e-07	< 1e-07	− 0.24	< 1e-07	< 1e-07
cg02145866	*MIR548G*	Body		− 0.29	< 1e-07	< 1e-07	− 0.30	< 1e-07	< 1e-07
cg26664528	*MIR548H4*	Body	Shore	− 0.28	< 1e-07	< 1e-07	NA	NA	NA
cg11539052	*MIR548N*	Body	Shore	− 0.20	< 1e-07	< 1e-07	NA	NA	NA
cg05966699	*MIR567*	TSS1500		− 0.16	< 1e-07	< 1e-07	− 0.24	< 1e-07	< 1e-07
cg06277638	*MIR570*	TSS1500		− 0.17	< 1e-07	< 1e-07	NA	NA	NA
cg09031823	*MIR575*	TSS1500		− 0.17	< 1e-07	7e-04	− 0.25	< 1e-07	< 1e-07
cg18948646	*MIR575*	TSS200		− 0.16	< 1e-07	< 1e-07	− 0.13	< 1e-07	< 1e-07
cg26620021	*MIR641*	TSS1500	Shore	− 0.28	< 1e-07	< 1e-07	NA	NA	NA
cg11035122	*MIR758*	TSS1500		− 0.15	< 1e-07	2e-06	− 0.18	< 1e-07	< 1e-07
cg02558026	*MIR762*	TSS1500	Shore	− 0.20	< 1e-07	< 1e-07	− 0.24	< 1e-07	< 1e-07
cg13204193	*MIR942*	Body		0.19	< 1e-07	< 1e-07	NA	NA	NA
cg01940297	*MIR1207*	TSS1500		0.16	< 1e-07	3e-06	0.25	< 1e-07	< 1e-07
cg25037841	*MIR1286*	TSS1500		0.15	< 1e-07	< 1e-07	0.18	< 1e-07	< 1e-07
cg25591377	*MIR1288*	TSS1500		− 0.27	< 1e-07	< 1e-07	− 0.24	< 1e-07	< 1e-07
cg02746110	*MIR1301*	TSS1500		0.16	< 1e-07	4e-07	0.18	< 1e-07	< 1e-07

*NA* probes not available in the TCGA processed data (level 3); *P Adj* Bonferroni *P* value; *FDR* false discovery ratio; *TSS* transcription start sites; *Shelf*, 2–4 kb from CpG island; *Shore*, until 2 kb from CpG island

### Impact of disrupted DNA methylation in miRNA expression

To identify alterations in the expression levels of miRNA genes in PTC, we assessed miRNA sequencing data from TCGA, revealing 58 miRNAs (27 up and 31 down expressed) (Additional file 2: Table S1). Only *MIR21* (hsa-miR-21-5p) and *MIR146B* (hsa-miR-146b-5p and hsa-miR-146b-3p) showed differential methylation and expression (hypomethylation with increased expression). The integrative analysis revealed highly significant negative correlation between methylation and miRNAs expression (Additional file 2: Table S2).

### Alterations in mRNAs targeted by miRNAs potentially regulated by methylation

The mRNA data analysis from TCGA revealed 2432 differentially expressed coding transcripts in PTC (Additional file 2: Table S3), 452 of them were considered as targets of the miRNAs affected by DNA methylation. Among these, 250, 243, and 189 mRNAs are predicted to interact with hsa-miR-146b-3p, hsa-miR-146b-5p, or hsa-miR-21-5p, respectively (681 miRNA-mRNA predicted interactions with significant negative correlation) (Additional file 2: Table S4).

### MicroRNAs and target genes associated with poor prognostic features

The DNA methylation levels of *MIR146B* and *MIR21*, expression of the miRNAs (*hsa-miR-146b-5p*, *hsa-miR-146b-3p*, and *hsa-miR-21*), and their target transcripts (452 mRNAs from the integrative analysis) were compared with the clinical-pathological findings (TCGA dataset). Hypomethylation and increased expression of *MIR146B*, as well as decreased target genes expression, were significantly associated with features related to poor prognosis (advanced clinical stage, lymph node metastasis, and extrathyroidal extension) and *BRAF* mutation (Additional file 2: Table S5).

### Data confirmation by quantitative bisulfite pyrosequencing and RT-qPCR

**Fig. 2:**
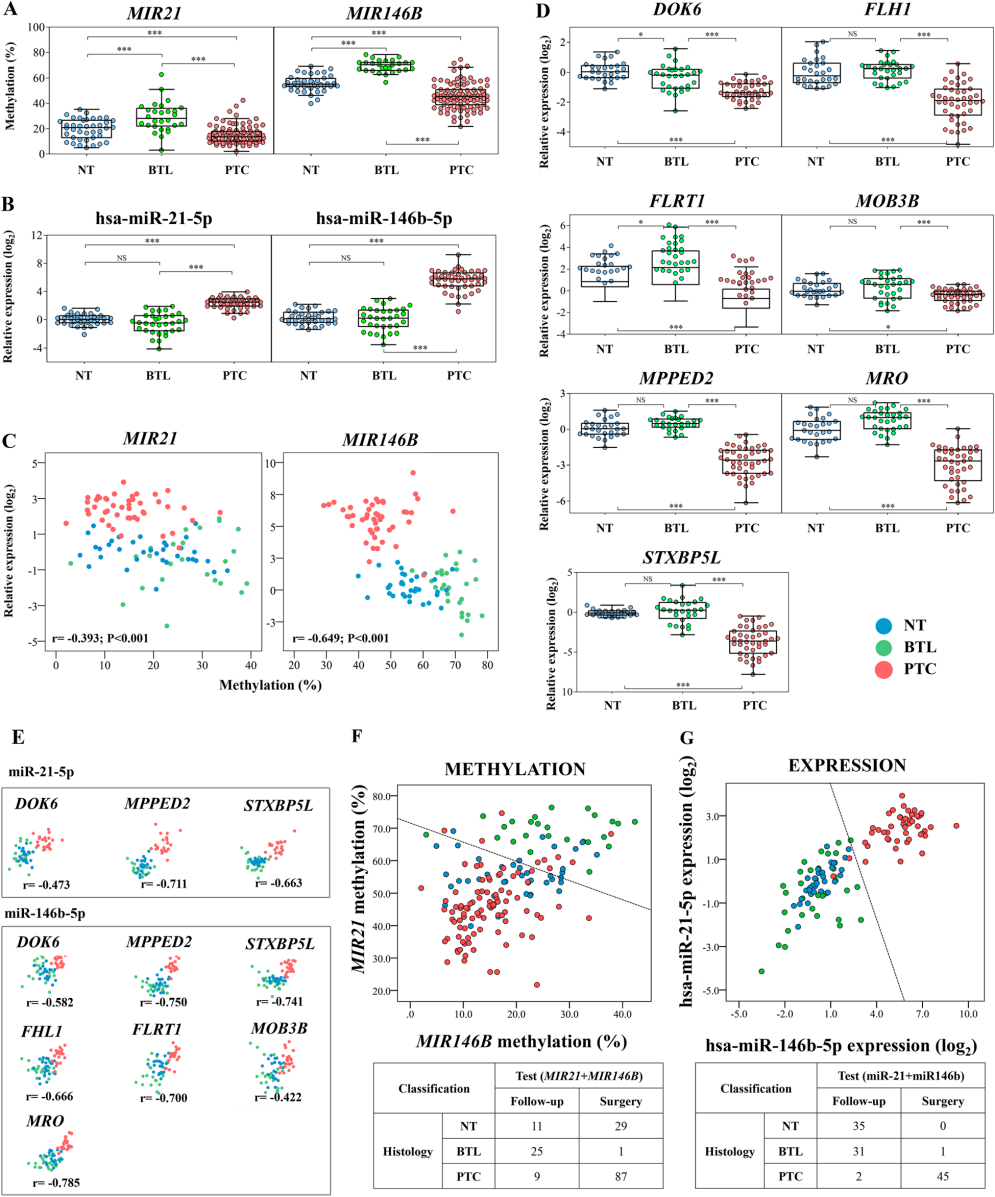
Quantitative bisulfite pyrosequencing and RT-qPCR confirmed the data from the large-scale analysis. *MIR21* and *MIR146B* were significantly hypomethylated (**a**) and overexpressed (**b**) in PTC compared to non-neoplastic tissues (NT and BTL). **c** Significantly negative correlation between methylation and expression of *MIR21* and *MIR146B* was observed. **d** Five miRNA-target transcripts showed lower expression in PTC compared to NT and BTL. No significant differences were found between NT and BTL, in exception of *DOK6* and *FLRT1* genes*.* **e** All target transcripts demonstrated significant negative correlation with their miRNA regulators (*P* < 0.001 to all genes). Scatterplot representation of *MIR21* and *MIR146B* methylation (**f**) and expression (**g**) levels. A diagnostic classifier (dashed line) was designed to distinguish PTC from BTL using Fisher’s linear discriminant analysis. The classification performance of the methylation and miRNA-based classifier is illustrated. Seven PTC and three BTL were excluded from the methylation diagnostic classifier (low pyrosequencing quality was observed for at least one of the miRNAs). NS, not significant; *P < 0.05; ***P < 0.001 (ANOVA followed by Tukey test). PTC, papillary thyroid carcinoma (red); NT, non-neoplastic thyroid tissue (blue); BTL, benign thyroid lesions (green); *r*, Pearson’s correlation coefficient; *P*, *p* value obtained by Pearson’s correlation test

Hypomethylation and overexpression of *MIR21* and *MIR146B* in PTC compared with NT and BTL were confirmed using quantitative bisulfite pyrosequencing and RT-qPCR, respectively (Fig. 2a, b). An additional analysis of PTC compared with NT samples from the same patients (18 matched samples for methylation and 17 for miRNA expression) also corroborated these findings (Additional file 1: Figure S2). A negative correlation between the methylation pattern and expression of *MIR21* (*r* = − 0.393; *P* < 0.001) and *MIR146B* (*r* = − 0.649; *P* < 0.001) was also observed (Fig. 2c).

Seven target genes selected for RT-qPCR evaluation (*DOK6*, *FHL1*, *FLRT1*, *MOB3B*, *MPPED2*, *MRO*, and *STXBP5L*) showed decreased expression in PTC compared to NT and BTL (Fig. 2d). The interactions among hsa-miR-146b-5p, hsa-miR-21-5p, and the seven targets, found in the integrative analysis using the TCGA dataset, supported the findings obtained in the RT-qPCR analysis (Fig. 2e).

The pyrosequencing and RT-qPCR results were also confronted with clinical-pathological features and *BRAF* mutation (observed in 59% of the PTC). A significant association was observed between lower methylation and higher expression levels of *MIR146B* and *MIR21* with *BRAF* mutation (Additional file 1: Figure S3).

### Development of a diagnostic tool in thyroid cancer

The DNA methylation (pyrosequencing) and expression (RT-qPCR) levels of *MIR21* and *MIR146B* were tested as a potential tool to discriminate malignant (PTC) from benign thyroid lesions (BTL). The combination of methylation values of both miRNAs allowed the discrimination of 87 out of 96 PTC and 25 out of 26 BTL (91% sensitivity and 96% specificity) (Fig. 2f). The hsa-miR-21-5p and hsa-miR-146b-5p relative expression was more efficient than methylation to classify correctly 45 of 47 PTC and 31 of 32 BTL (96% sensitivity and 97% specificity) (Fig. 2g). Curiously, the miRNA expression test correctly classified all NT samples as non-malignant (35 of 35), while the methylation test categorized 29 of 40 NT samples (73%) as malignant.

### Global demethylation-induced *MIR146*B expression in TPC1 cell line

**Fig. 3:**
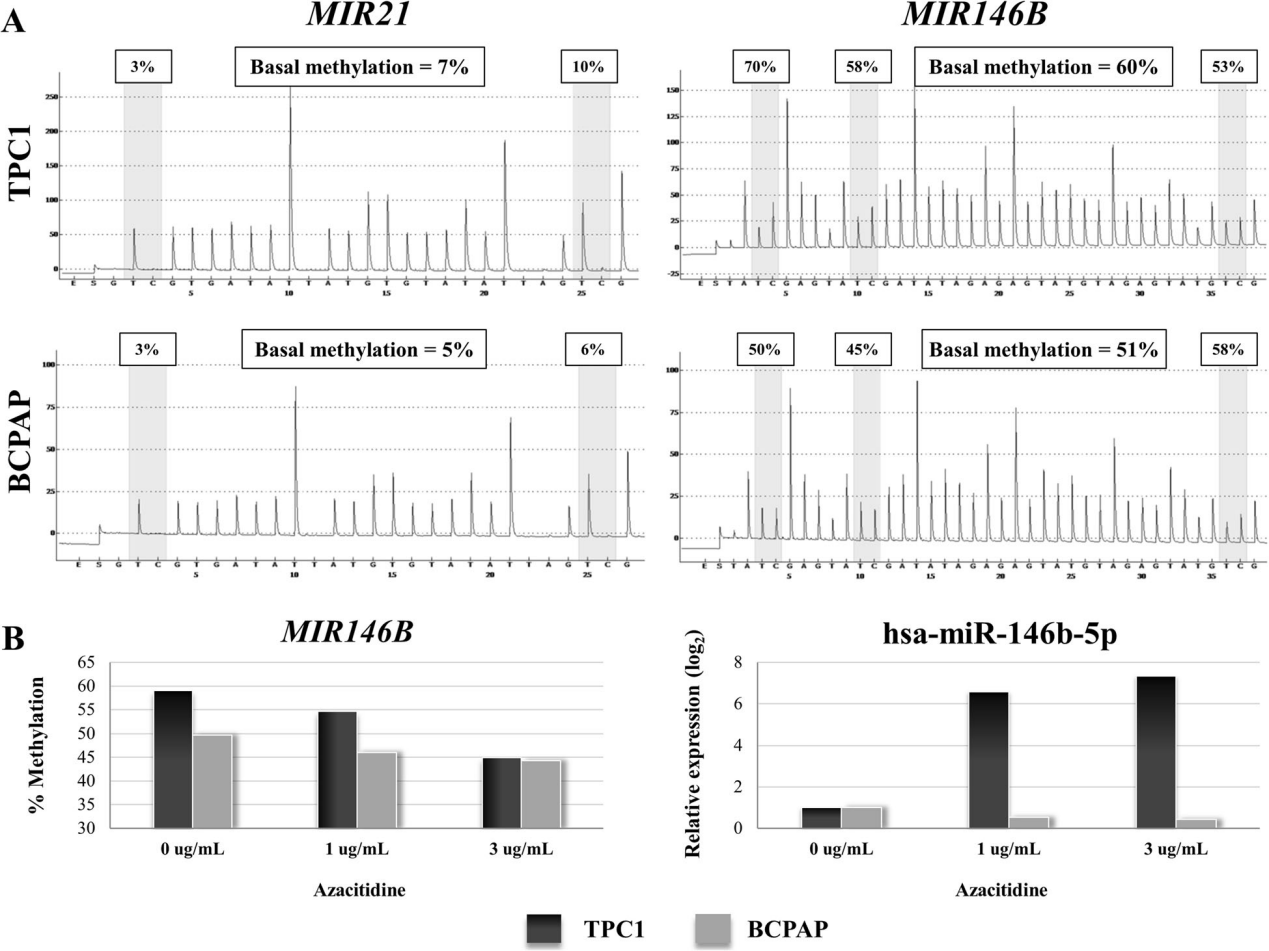
Demethylation assays in thyroid cancer cell lines (TPC1 and BCPAP). **a** Pyrogram showing the percentage of basal methylation of *MIR21* (unmethylated in both cell lines) and *MIR146B* (> 50% methylation in both cell lines). **b** The demethylation in the mapped region of *MIR146B* was demonstrated after 5-Aza-dC treatment in TPC1 and BCPAP cell lines. BCPAP cell line had no changes in hsa-miR-146b-5p expression and TPC1 cell line presented seven-fold increased expression levels after the induced demethylation

Firstly, we investigated the basal methylation of two PTC cell lines (TPC1 and BCPAP) to study hypomethylated CpGs. Whereas *MIR146B* showed high methylation levels in both cell lines (51–60% of methylated alleles), *MIR21* was completely unmethylated even at basal level (5–7% of methylated alleles), rendering no change in miR expression after 5-Aza-dC treatment (Fig. 3a). Loss of global methylation (*AlR1Sat* and *AluYB*8 repetitive regions) was confirmed after treatment with 5-Aza-dC. Specific loss of methylation in CpGs mapped in the *MIR146B* gene and an increased expression level of the hsa-miR-146b-5p in the TPC1 cell line was also observed (Fig. 3b).

No change in miR expression.

### Decreased expression levels of target mRNAs after miRNA mimic transfection in the TPC1 cell line

A transfection assay using the TPC1 cell line for each miRNA mimic (hsa-miR-146b-3p, hsa-miR-146b-5p and hsa-miR-21-5p) was performed followed by global transcriptomic analysis. From the 452 mRNAs highlighted by the integrative analysis (681 miRNA-mRNA interactions), 168 also showed decreased expression levels after transfection (188 miRNA-mRNA interactions). This strategy gives additional evidence that 136, 112, and 134 mRNA targets are regulated by hsa-miR-146b-3p, hsa-miR-146b-5p, and hsa-miR-21-5p, respectively (Additional file 2: Table S6). The in silico pathway analysis (IPA and KOBAS 3.0) including the mRNA target candidates revealed the involvement of canonical pathways related to neuronal system, thyroid function (mainly for hsa-miR-146b-3p targets), and MAPK/ERK signaling (mainly for hsa-miR-146b-5p targets) (Additional file 2: Tables S7 and S8).

## Discussion

Aberrant microRNA expression in thyroid neoplasia was previously reported [14, 15]; however, the mechanisms underlying the regulation of these miRNAs are poorly explored. As possible impact of DNA methylation was previously noticed [7]. In this study, we investigated the methylation profiles of PTC and NT in genes encoding miRNA using a high coverage platform (Illumina 450 k). Although the use of whole genome bisulfite sequencing would encompass more miRNA gene regions, an advantage of our strategy was the inclusion of 565 PTC and 106 NT evaluated by the same platform (internal and external samples from TCGA), which strengthened our findings.

*MIR21* exhibited increased expression and decreased methylation levels in PTC compared to NT and BTL. *MIR21* overexpression was previously reported in thyroid cancer [16, 17]. The transcribed miRNA (hsa-miR-21) was one of the first oncomiR described and one of the most studied in several tumor types [18–20]. In prostate cancer cell lines, *MIR21* promoter hypermethylation resulted in its repressed expression [21]. Moreover, the 5-AZA-dC treatment stimulated the expression of this miRNA in prostate and ovary cancer cell lines [21, 22]. However, these studies were restricted to cell lines, and tumor samples were not evaluated to confirm the *MIR21* methylation pattern.

Similar to *MIR21*, *MIR146B* presented hypomethylation and increased expression levels in PTC. This miRNA was also previously described as overexpressed in PTC [7, 14, 15]. Contrarily, the hypermethylation and down-expression of *MIR146B* were reported in diffuse and anaplastic astrocytomas, gliomas, and breast cancer being the 5-AZA-dC treatment capable to induce the *MIR146B* expression [23, 24]. Taken together, these findings give evidences of the regulatory role of *MIR146B* in different tumor types, being able to act as tumor suppressor or oncomiR, depending on the context of altered regulatory pathways in each tumor type [25–28].

The identification of miRNAs target-genes is crucial to understand the regulatory mechanisms involved in thyroid cancer cells. In this context, 452 target transcripts were unveiled by the miRNA-mRNA integrative analysis (TCGA dataset). Interestingly, 168 of these 452 putative mRNA targets also exhibited decreased expression levels after the individualized mimic transfection assays using these three miRNAs (hsa-miR-146b-3p, hsa-miR-146b-5p, or hsa-miR-21-5p). Although the cell lines are very useful models, gene expression profiles are not identical with those found in primary tumor tissues. Differences based on epigenetic reprogramming have been reported as a causal effect of the in vitro conditions [29]. In addition, the induction of a single miRNA can influence the expression of many transcriptional factors, which enhances the complexity of the transcriptome regulation network and makes the outcome less predictable [30].

Seven selected transcripts were confirmed as having decreased expression levels in PTC compared to NT and BLT. Among the selected genes, *MPPED2*, *MRO*, *STXBP5L*, *FHL1*, and *FLRT1* were previously reported as down-regulated in PTC [31, 32]. In prostate [33], stomach [34], and breast cancer [35], *DOK6* and *MOB3B* were reported as putative tumor suppressor genes. In follicular tissues of the thyroid gland, these genes could have similar function of tumor suppressors.

We also investigated the association of miRNAs methylation and expression levels of the target-genes with clinical and pathological features of poor prognosis and *BRAF* mutation. Increased expression levels of *MIR146B* were related with advanced stage, tumor size, extrathyroidal extension, and *BRAF* mutation, as previously reported [36–39]. In our initial analysis using the TCGA cohort, *MIR21* and *MIR146B* hypomethylation and their increased expression levels, as well as decreased levels of several target-genes, were suggested as associated with poor prognosis features. Nevertheless, a clear distinction of the molecular profiles was confirmed using pyrosequencing and RT-qPCR analysis in our *BRAF*-mutated cases. The association between global DNA methylation and *BRAF* mutation in PTC has already been extensively explored, and a global hypomethylation in tumors harboring *BRAF* V600E was reported [8, 40, 41]. The mechanisms possibly involved in the epigenetic control of genes by the *BRAF* mutation have been recently explored (42–44). In melanoma cells, the expression of 59 hypermethylated genes in the *BRAF* V600E knockdown were down-expressed suggesting that in mutated cells these genes were hypomethylated and over-expressed [42]. According to the authors, aberrant *EZH2* (histone methyltransferase) and *DNMT1* (DNA methyltransferase 1) expression were affected by the *BRAF* mutation [42]. A similar study performed by the same group was reported in two PTC cell lines (BCPAP and OCUT1), showing that *BRAF* mutation had an impact in the methylation and the expression of several genes, including *HMGB2* and *FDG1* [43]*.* A recent study demonstrated that the MAPK/ERK signaling pathway activated by the *BRAF* mutation was able to induce epigenetic aberrations by H3K27me3 and *MYC* [44]. Therefore, if *BRAF* mutation can trigger the epigenetic alterations in coding genes, the same effect is expected to occur in miRNAs.

*MIR21* and *MIR146B* methylation and expression analysis revealed high sensitivity (91% and 96%, respectively) and specificity (96% and 97%, respectively) to distinguish PTC from benign lesions. Although the methylation classifier failed in distinguishing PTC from NT (73% of the NT samples were classified as malignant), the miRNA expression classifier categorized all NT as “non-malignant.” These results suggest that our methylation markers could detect cells in the preliminary steps of malignant transformation (the non-neoplastic samples were obtained from the surrounding thyroid tissue from PTC patients). Epigenetic events are known to anticipate the phenotypic manifestation of malignancy [45, 46].

Considering the similarities observed in the classifiers for molecular diagnosis in thyroid nodules and the high complexity of the methylation assays, miRNAs expression analysis is more easily applicable in the clinical routine. Two recent studies [47, 48] reported the clinical validation of assays based on miRNA expression (Rosetta Genomics, Philadelphia, PA, USA). In these studies, a set of 24 miRNAs were evaluated (among them, hsa-miR-146b-5p) by RT-qPCR in thyroid nodules showing indeterminate cytology. First, an analytical validation was conducted to ensure the test robustness, proving the feasibility of the assay in fine-needle aspiration smears (*N* = 576 nodules) [47]. Subsequently, a classifier was developed to identify nodules as benign or suspect (*N* = 189 cases) showing 98% of sensitivity and 78% of specificity [48]. Despite our malignant cohort was only represented by surgical specimens of PTC, we achieved a similar sensitivity and higher specificity.

Although most of studies focused in the discovery of tumor suppressor miRNAs repressed by epigenetic mechanisms [49, 50], only hypomethylated miRNA-encoding genes were detected in our study. Cell lines treated with demethylating agents, as 5-Aza-dC, is widely used to demonstrate a direct regulation by methylation [51]. Even though *MIR21* and *MIR146B* were hypomethylated in PTC, the basal CpG methylation found in *MIR146B* was relatively high in two cell lines evaluated (TPC1 and BCPAP), allowing a functional assay using 5-Aza-dC. The increased expression of *MIR146B* after the treatment infers an association with the methylation loss in the miRNA gene promoter.

The role of hsa-mir-146b-5p in proliferation, migration, and invasiveness of thyroid cancer was previously investigated by others using mimics-miR transfection assays in thyroid cell lines [52, 53]. The functional role of hsa-mir-146b-5p in thyroid gland oncogenesis and its association with PTC aggressiveness was reported as involved with the down-regulation of *PTEN* and E-cadherin [53]. Likewise, it was previously described that inducing hsa-miR-21-5p in TPC1 cell line by plasmid transfection (pEZX-eGFP-miRNA-21) resulted in increased cell proliferation and invasion, and inhibited apoptosis, possibly mediated by *PDCD4* repression [16].

## Conclusions

The mechanism underlying the overexpression of *MIR21* and *MIR146B* due to DNA methylation loss in PTC was explored in our study. We used, for the first time, small-scale (RT-qPCR and pyrosequencing) analysis and functional assays to corroborate the findings originated from large-scale screening in a large cohort of cases. The interconnections between these epigenetic events are potentially responsible for many deregulations in the thyroid transcriptome leading to cancer development. DNA methylation and expression levels of these miRNA-encoding genes were demonstrated as suitable PTC diagnostic markers. Moreover, the understanding of the mechanisms of upregulation of these oncomiRs in thyroid malignancies creates opportunities to develop miRNA-targeting therapies.

## Methods

### Sample population

Fifty matched PTC and NT samples from our previous DNA methylation profiling studies were re-evaluated as a discovery set. In addition, a confirmatory subset of 103 PTC, 40 NT, and 32 BTL (benign thyroid lesions: 14 follicular adenomas, 17 goiters, and 1 lymphocytic thyroiditis) snap-frozen tissues were obtained retrospectively from patients treated at A.C. Camargo Cancer Center, São Paulo, SP, Brazil. The Additional file 2: Table S9 summarizes the clinical features of PTC patients. Nucleic acid isolation and *BRAF* mutation genotyping are detailed in Additional file 3: Supplementary Methods.

### Global DNA methylation analysis

DNA methylation profiling were obtained from the 50 paired PTC and NT samples using the Infinium® Human Methylation450 BeadChip Platform (Illumina, San Diego, CA, USA). The data were retrieved from previously generated studies from our group [8, 10] and are available in the Gene Expression Omnibus database (GSE86961 and GSE97466). CpG probes differentially methylated in PTC compared to NT were detected using limma package [54] with adjusted *P* value (Bonferroni) < 0.05 and delta-beta (Δβ) > 0.15 or < − 0.15. Quality controls and pre-processing data are detailed in Additional file 3: Supplementary Methods.

### Integrative analysis using TCGA database

The available TCGA clinical and molecular data were retrieved using UCSC Xena (https://xenabrowser.net/datapages/—accessed in February 2018). DNA methylation data from 515 PTC and 56 NT (Infinium® Human Methylation450 BeadChip) from TCGA dataset were used to confirm the CpG probes differentially methylated identified in our study (*t* test adjusted *P* < 0.05, Δβ > 0.1, or < − 0.1). Two strategies using integrative analysis were developed: (i) CpG probes differentially methylated in both cohorts were compared to miRNAs expression from TCGA (miRNASeq IlluminaHiSeq, 509 PTC and 59 NT), and (ii) miRNAs differentially expressed were contrasted with target-genes expression using the TCGA data (RNASeqV2 IlluminaHiSeq) in 505 PTC versus 59 NT (*t* test adjusted *P* < 0.05; fold change FC > 2 for both miRNA and mRNA). As different prediction methods are generally uncorrelated [55], miRNA target prediction was carried out with miRWalk 2.0 tool (http://zmf.umm.uni-heidelberg.de/apps/zmf/mirwalk2/), considering predicted interactions in at least two of four selected algorithms (miRWalk, miRanda, RNAhybrid, and Targetscan). Predictions were based on interactions between miRNA seed sequences (starting from the first position) and the 3′UTR region of the target mRNA. Pearson’s correlation test was applied to investigate negatively correlated predicted interactions in PTC (*P* < 0.05).

### DNA methylation analysis in miRNAs genes by pyrosequencing

To confirm the global DNA methylation results, 500 ng of genomic DNA samples were converted by sodium bisulfite using the EZ DNA Methylation Gold kit (Zymo, Irvine, CA, USA), according to the manufacturer’s recommendations. Only independent samples (103 PTC, 29 BTL, and 40 NT) from the previous global methylation analysis [8, 10] were included. Forward and reverse biotinylated primers (Sigma, Darmstadt, Germany) were used to amplify the region of interest (Additional file 2: Table S10 and Additional file 1: Figure S4). Two probes representative of *MIR146B* and *MIR21* were selected, presenting the highest negative correlation with the corresponding miRNA expression. Primer design and PCR conditions are detailed in Additional file 3: Supplementary Methods. Pyrosequencing (PyroMark Q24 system, Qiagen) was performed including methylated and unmethylated DNA controls according to the manufacturer’s instructions (Zymo Research, Irvine, CA, USA).

### MicroRNAs and target mRNAs expression analysis by RT-qPCR

Expression of miRNAs (hsa-miR-21-5p and hsa-miR-146b-5p) and mRNAs (*MPPED2*, *STXBP5L*, *MRO*, *FHL1*, *FLRT1*, *DOK6*, and *MOB3B*) were performed by RT-qPCR (detailed in Additional file 3: Supplementary Methods) using TaqMan® and SYBR® Green detection system, respectively (Applied Biosystems, CA, USA). The hsa-miR-146b-5p and hsa-miR-146b-3p showed redundant expression in the miRNA sequencing results using the TCGA dataset (PTC = 510, Pearson’s *r* = 0.970, Pearson’s *P* not computable). Based on these findings, we selected only hsa-miR-146b-5p to be evaluated by RT-qPCR (most prevalent mature form).

Seven of 452 target transcripts detected in the integrative analysis were selected according to the negative correlation coefficient values, expression levels in normal thyroid tissues (available at https://www.ncbi.nlm.nih.gov/gene), higher FC, and significant clinicopathological variables associated with poor prognosis (obtained from TCGA) (Additional file 2: Table S11). According to these parameters, all seven transcripts are targets of miR-146b-5p, and three of them (*DOK6*, *MPPED2*, and *STXBP5L*) are targets of miR-21-5p. The mRNA primer sequences are described in Additional file 2: Table S12.

For miRNA analysis, TaqMan® microRNA Reverse Transcription Kit (Applied Biosystems) and TaqMan® microRNA Assays (IDs: 000397 and 001097, Applied Biosystems) were used according to the manufacturer’s instructions. miRNA normalization was performed using *RNU44* (ID 001094) and *RNU48* (ID 001006) [36, 56]. The highly stable references, *EIF2B1* and *PUM1*, were employed for mRNA normalization, as previously described [31]. The method proposed by Pfaffl (2001) [57] was used for normalization with a geometric mean of reference tests and efficiency equal to 100%.

### Global demethylation assay in PTC cell lines

The human thyroid cancer cell lines TPC1 (*BRAF* wild type, received from Janete M Cerutti, Federal University of São Paulo, Brazil) and BCPAP (*BRAF* V600E, received from Edna T. Kimura, University of São Paulo, Brazil) were in vitro cultured in RPMI (Gibco, Grand Island, NY, USA) and DMEM/F-10 medium (Gibco), respectively, supplemented with 10% fetal bovine serum, 1% streptomycin (Gibco), and 1% penicillin (Gibco). Global demethylation assays were performed using 5-Aza-dC (Sigma, Darmstadt, Germany) at 1 μM and 3 μM determined by cell viability assays (detailed in Additional file 3: Supplementary Methods). Loss of methylation after treatment was confirmed by pyrosequencing using AluYB8 and AlR1Sat primer pairs, as described by Choi et al. (2009) [58]. Basal methylation of *MIR21* and *MIR146B* regions and the corresponding mature miRNAs expression in the cell lines upon treatment were evaluated as described above. The samples treated with 5-Aza-dC were compared to vehicle using three replicates (independent assays) for each cell line.

### MicroRNA mimics transfection in the TPC1 cell line

Due to the increased expression of *MIR146B* after azacitidine treatment in TPC1, this cell line was chosen to conduct the transfection experiments. The cells were seeded 24 h prior to the transfection in 6-well plates with 200,000 cells per well in the medium specific to the cell lines, without supplemented antibiotics. The transfection reagent Lipofectamine RNAiMAX (Invitrogen) was prepared in Opti-MEM medium (Invitrogen), as recommended by the manufacturer. The mirVana miRNA mimics (Invitrogen) hsa-miR-146b-3p, hsa-miR-146b-5p, and hsa-miR-21-5p were dissolved to the relevant concentrations in Opti-MEM medium. The diluted transfection reagent and mimics (final concentration of 30 nM) were then mixed and incubated at room temperature for 5 min. Afterwards, the complexes were added to each well containing cells and Opti-MEM medium. Negative control mimics (30 nM) and a mock control were included in the transfection experiments. The cells were incubated for 48 h before being harvested in trypsin followed by total RNA extraction. The mature miRNAs were reversely transcribed with targeted primers. Moreover, successful transfection was confirmed by RT-qPCR (> 100 increased expression levels for the three miRNAs).

### Large-scale transcriptomic analysis after miRNA transfection

The RNA from mimics and control assays was amplified (200 ng), labeled, and hybridized using the Clariom D platform (Affymetrix, Santa Clara, CA, USA) following the manufacturer’s instructions. Two biological replicates were included for each miRNA tested individually. The arrays hybridization was performed in a GeneChip® Hybridization Oven 645 (Affymetrix) and scanned using the GeneChip Scanner 3000 (Affymetrix). The data were analyzed using the Affymetrix Transcriptome Analysis Console software (v. 3.1.0.5) and normalized by the Robust Multiarray Average module. The analysis was focused in the target mRNAs found in the integrative analysis and specifically in transcripts down-expressed (in both duplicates) after the transfection with the mimics.

### In silico canonical pathway analysis

Protein-encoding genes predicted as target of the miRNAs potentially regulated by DNA methylation, found in the integrative analysis and down-expressed after the mimic transfection, were submitted to canonical pathway evaluation using the Ingenuity Pathway Analysis (IPA v2.1, Ingenuity Systems) and KOBAS 3.0 software (http://kobas.cbi.pku.edu.cn/).

### Statistical analysis

The SPSS v. 21.0 (Statistics Packet for Social Sciences, Chicago, IL, USA) and BRB Array Tools v. 4.4.0 were used for statistical analysis. Graphical representations were implemented by GraphPad Prism v.5.0 (GraphPad Software Inc., La Jolla, CA, USA). The methylation, miRNAs, and target gene expression from TCGA were compared with clinical and pathological data and *BRAF* mutation using *t* test (*P* < 0.001; FDR < 5%; |Δβ| > 0.10 or FC > 1.5). Pyrosequencing (percentage of methylation in each CpG) and RT-qPCR data (miRNAs and mRNAs relative expression) were evaluated by parametric tests (paired and unpaired *t* test, ANOVA with Turkey’s post hoc, and Pearson’s correlation test). The null hypothesis was rejected when the two-tailed *P* value was < 0.05. Fisher discriminant analysis was used to construct diagnostic classifier algorithms.

## Additional files

Additional file 1: **Figure S1.** Supervised hierarchical clustering analysis heatmaps comprising 42 probes of miRNAs identified in both, internal (**A**) and TCGA (**B**) data. The clusters highlighted in red demonstrate enrichment for PTC samples and in blue for NT samples. PTC: papillary thyroid carcinoma; NT: non-neoplastic thyroid tissue. **Figure S2.** Matched papillary thyroid carcinomas (PTC) compared with non-neoplastic thyroid tissue (NT) samples showed hypomethylation and miRNA increased expression of *MIR21* and *MIR146B* in PTC (***P < 0.001; paired *t* test). **Figure S3.** Methylation (**A**) and expression (**B**) analysis of *MIR21* and *MIR146B* according to *BRAF* mutation status. PTC, papillary thyroid carcinoma; *BRAF*WT, *BRAF* wild type; *BRAF*V600E, positive for *BRAF* mutation. *P < 0.05; **P < 0.01; ***P < 0.001 (Student’s *t* test). **Figure S4.** Location of the probes covering the *MIR21* (**A**) and *MIR146B* (**B**) at chromosomes 17 and 10, respectively. The probes highlighted in orange were selected for pyrosequencing confirmation. (DOCX 5782 kb)

Additional file 2: **Table S1.** Identification of 58 differentially expressed microRNAs detected in the comparison between PTC and NT from the TCGA database. **Table S2.** Integration between DNA methylation probes and the corresponding miRNA expression. **Table S3.** Differentially expressed genes in PTC compared to NT from TCGA database. **Table S4.** MicroRNA and target-mRNA interactions obtained by the integrative analysis (TCGA database), comprising the miRNAs potentially regulated by DNA methylation. **Table S5.** Comparison of miRNA genes methylation, miRNA, and target gene expression with clinical features (clinical stage, extrathyroidal extension, node metastasis and *BRAF* mutation) using the TCGA database. **Table S6.** List of 452 target transcripts of miRNA potentially regulated by methylation after mimic transfection in TPC1 cell line. **Table S7.** Significant pathways (*P* value< 0.05) enriched by target genes of hsa-miR-21-5p and hsa-miR-146b (5p and 3p) identified by IPA. **Table S8.** Significant pathways (*P* value< 0.01) enriched by target genes of hsa-miR-21-5p and hsa-miR-146b (5p and 3p) identified by KOBAS 3.0 (http://kobas.cbi.pku.edu.cn). **Table S9.** Clinical-pathological characteristics of the patients included in the study. **Table S10.** Primer sequences, amplicon size, number of CpGs flanked in the amplification and PCR temperature conditions. **Table S11.** Selection criteria of the miRNA targets selected for RT-qPCR validation. Table S12. Characteristics of the primers used in the transcripts expression level analyses by RT-qPCR. (XLSX 295 kb)

Additional file 3: Supplementary methods. (DOCX 29 kb)

## Abbreviations

5-Aza-dC: 5-aza-deoxycytidine
BTL: Benign thyroid lesions
CpG: 5′-deoxycytosine-phosphate-deoxyguanosine-3
IPA: Ingenuity pathways analysis
KOBAS: KEEG orthology based annotation system
miRNA: MicroRNA
mRNA: Messenger RNA
NT: Non-neoplastic adjacent tissues
PTC: Papillary thyroid carcinoma
RT-qPCR: Reverse transcription quantitative polymerase chain reaction
TCGA: The Cancer Genome Atlas
