# Supplementary material for: Loss of DNA methylation is related to increased expression of miR-21 and miR-146b in papillary thyroid carcinoma
Source: Clin Epigenetics. 2018 Nov 20;10:144. doi: 10.1186/s13148-018-0579-8 (PMC6245861; doi:10.1186/s13148-018-0579-8)
Supplement: Supplementary file 1 — Figure S1. Supervised hierarchical clustering analysis heatmaps comprising 42 probes of miRNAs identified in both, internal (A) and TCGA (B) data. The clusters highlighted in red demonstrate enrichment for PTC samples and in blue for NT samples. PTC: papillary thyroid carcinoma; NT: non-neoplastic thyroid tissue. Figure S2. Matched papillary thyroid carcinomas (PTC) compared with non-neoplastic thyroid tissue (NT) samples showed hypomethylation and miRNA increased expression of MIR21 and MIR146B in PTC (***P < 0.001; paired t test). Figure S3. Methylation (A) and expression (B) analysis of MIR21 and MIR146B according to BRAF mutation status. PTC, papillary thyroid carcinoma; BRAFWT, BRAF wild type; BRAFV600E, positive for BRAF mutation. *P < 0.05; **P < 0.01; ***P < 0.001 (Student’s t test). Figure S4. Location of the probes covering the MIR21 (A) and MIR146B (B) at chromosomes 17 and 10, respectively. The probes highlighted in orange were selected for pyrosequencing confirmation. (DOCX 5782 kb) [file 13148_2018_579_MOESM1_ESM.docx]

**SUPPLEMENTARY FIGURES**

***
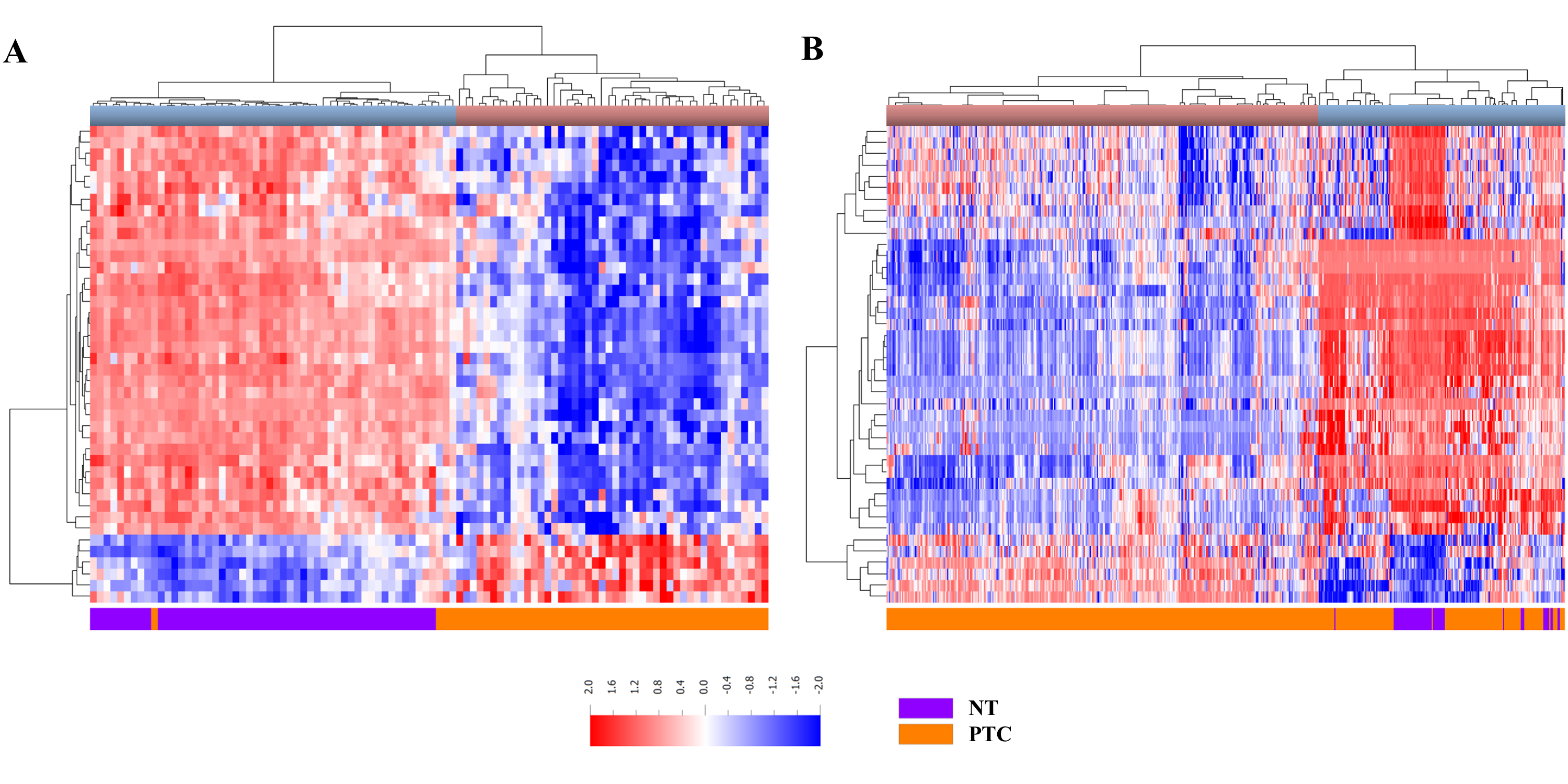
*Figure S1.** Supervised hierarchical clustering analysis heatmaps comprising 42 probes of miRNAs identified in both, internal (**A**) and TCGA (**B**) data. The clusters highlighted in red demonstrate enrichment for PTC samples and in blue for NT samples. PTC: papillary thyroid carcinoma; NT: non-neoplastic thyroid tissue.

**
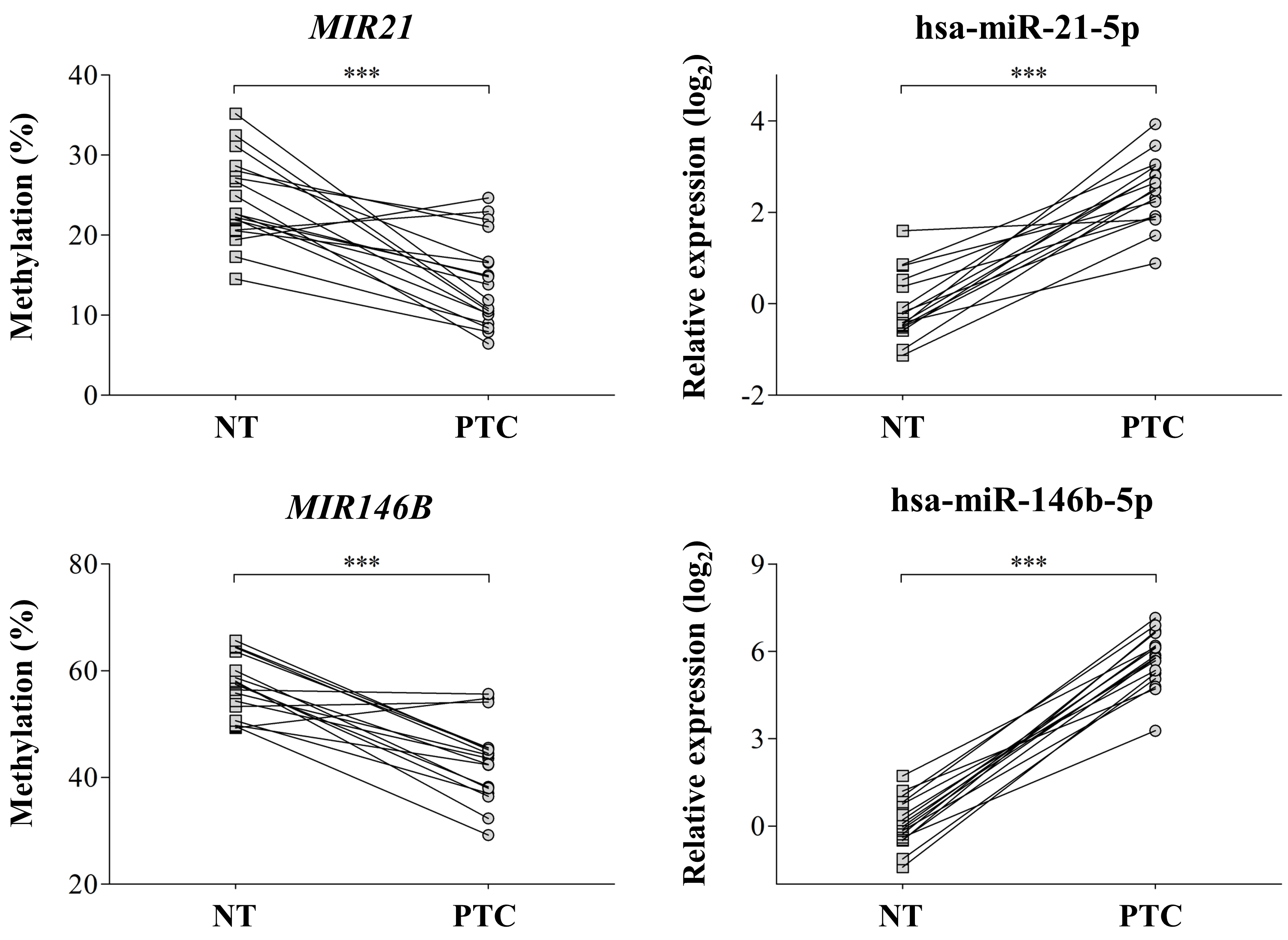
**

**Figure S2.** Matched papillary thyroid carcinomas (PTC) compared with non-neoplastic thyroid tissue (NT) samples showed hypomethylation and miRNA increased expression of *MIR21* and *MIR146B* in PTC (*** P< 0.001; paired t test).

**
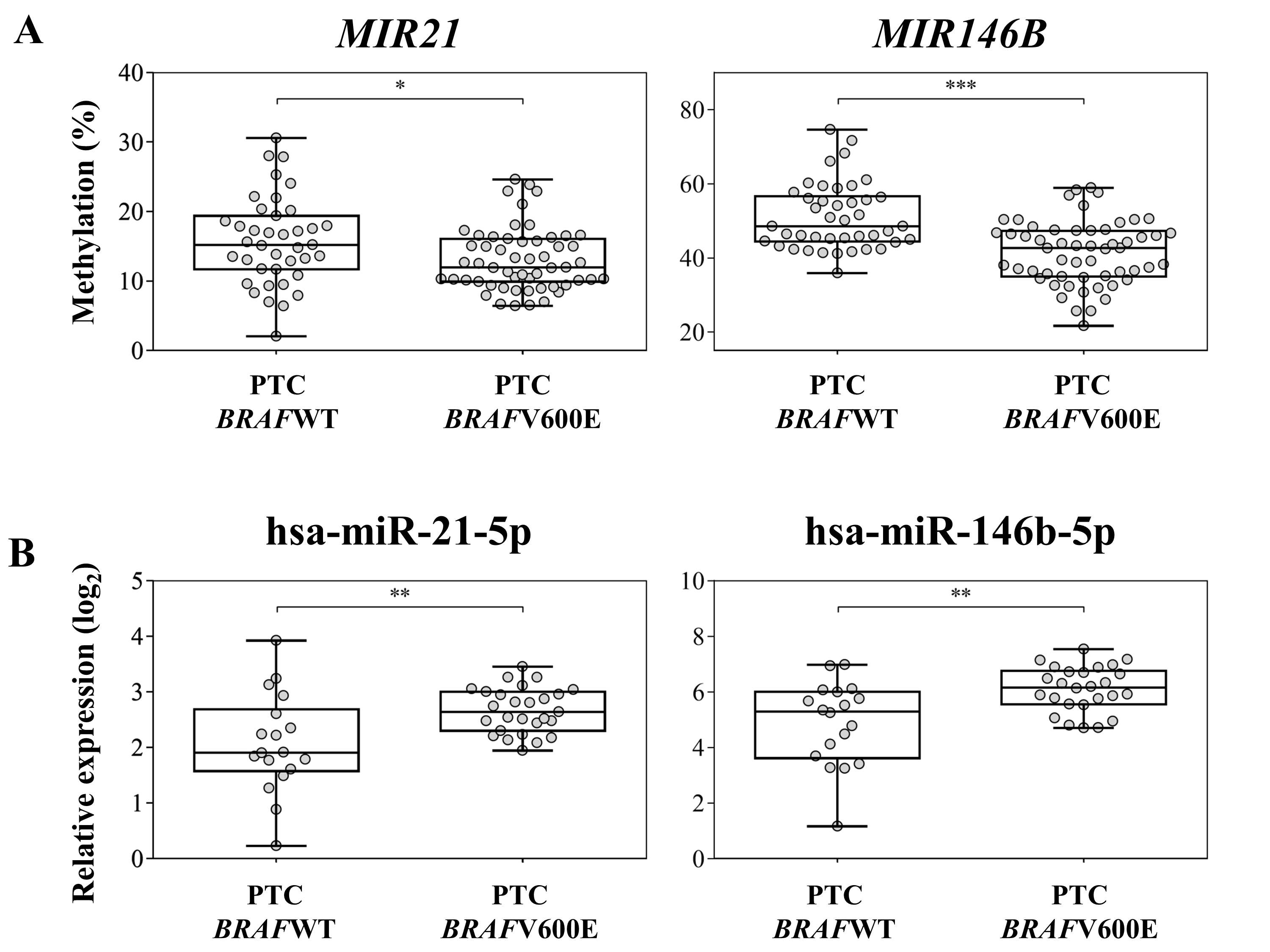
**

**Figure S3.** Methylation (**A**) and expression (**B**) analysis of *MIR21* and *MIR146B* according to *BRAF* mutation status. PTC: Papillary thyroid carcinoma; *BRAF*WT: *BRAF* wild type; *BRAF*V600E: positive for *BRAF* mutation. * P< 0.05; ** P< 0.01; *** P< 0.001 (Student t test).

**
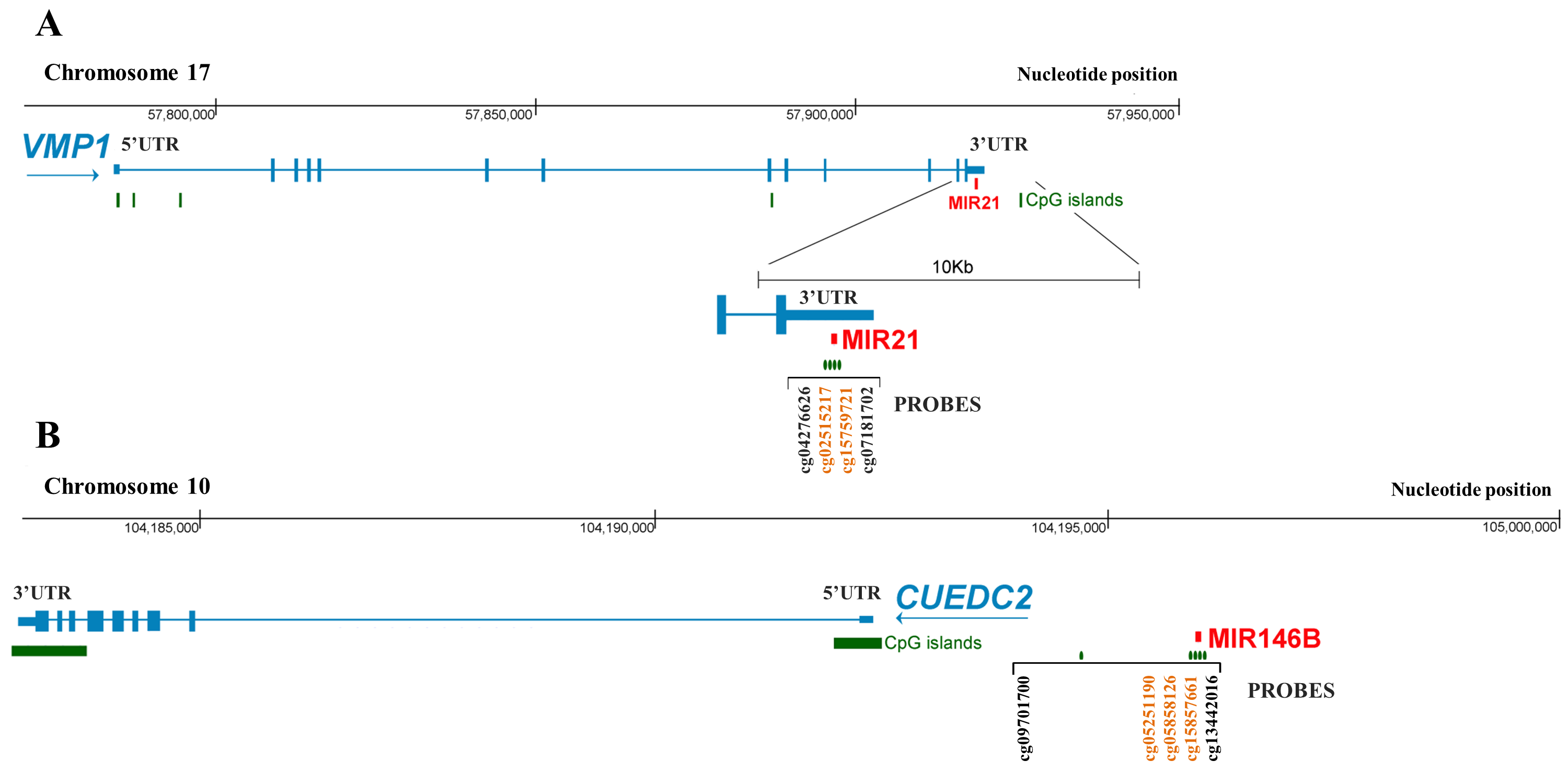
Figure S4.** Location of the probes covering the *MIR21* (**A**) and *MIR146B* (**B**) at chromosomes 17 and 10, respectively. The probes highlighted in orange were selected for pyrosequencing confirmation.
