# Supplementary material for: Loss of DNA methylation is related to increased expression of miR-21 and miR-146b in papillary thyroid carcinoma
Source: Clin Epigenetics. 2018 Nov 20;10:144. doi: 10.1186/s13148-018-0579-8 (PMC6245861; doi:10.1186/s13148-018-0579-8)
Supplement: Supplementary file 3 — Supplementary methods. (DOCX 29 kb) [file 13148_2018_579_MOESM3_ESM.docx]

**SUPPLEMENTARY METHODS**

**Nucleic acid extraction**

DNA isolation was based on the standard protocol using enzymatic degradation with proteinase K followed by purification with organic solvents (phenol/chloroform). RNA was extracted using TRIZOL™ (Invitrogen, Carlsbad, CA, USA) or miRNA Symphony kit (Qiagen, Valencia, CA, USA), according to the manufacturer's recommendations. DNA was quantified by Qubit® dsDNA BR Assay (Life Technologies, Carlsbad, CA, USA). The RNA quantity and quality were evaluated by NanoDrop (ND-1000 Spectrophotometer v.3.0.1, Labtrade) and Bioanalyzer (RNA 6000 NanoLabChip kit 2100 Agilent Technologies), respectively.

***BRAF* mutations analysis**

PTC samples were investigated for *BRAF* mutation using pyrosequencing method (PyroMark Q24, Qiagen, Valencia, USA). A total of 20ng of genomic DNA was used to amplify the sequences selected with forward and reverse biotinylated primers (ADS871, Epigendx, Worcester MA, England). Positive and negative controls to *BRAF* mutation (WiDr commercial cell line and human DNA unmethylated, Epitech, Qiagen) were included in the experiments. *BRAF* mutation was considered positive in cases having T-A transversion mutation at codon 600 and allele frequency of at least 10%.

**Quality control and global DNA methylation data processing**

The quality control of the raw data was performed using R program v.3.0.2 (https://www.r-project.org/) and the WateRmelon Bioconductor analysis package (Pidsley et al., 2013). Probes mapped on the X and Y chromosomes, SNPs (minor allele frequency > 5%), and sequences that co-hybridized into homologous alternative sequences (≥ 49 bases) were filtered out (Chen et al. 2013). Probes presenting *P* values > 0.05 and bead count <3 in at least 5% of the samples were also excluded. The color bias adjustment and data normalization between the samples were performed by the quantile method and between probes by the BMIQ (Beta-Mixture Quantile Normalization) method. Possible variations in the results generated by the position and time points of the samples in the arrays were corrected with batch effects analysis using the SVA package (Leek et al., 2012). Supervised hierarchical clustering analyses were performed using 1-minus correlation distance and complete linkage by BRB Array Tools v. 4.4.0 (http://linus.nci.nih.gov/BRB-ArrayTools.html).

**Primer design for PCR prior to pyrosequencing**

The primers designed to evaluate two genes encoding miRNAs (*MIR146B* and *MIR21*) followed the criteria: (i) primers size ranging from 18 to 24 bases, (ii) without 3 guanine (G) or cytosine (C) in 5’ end, (iii) same amount of C and G in the forward and reverse primers, (iv) the presence of at least 50% of C and G content, (v) amplicon smaller than 300 base pairs, and (vi) melting temperature between 55-62°C. PCR was performed using 20ng of converted DNA and 200nM of each primer. The cycling conditions were: 90°C for 15 minutes, 50 cycles at 95°C for 30 seconds, 54-55°C for 30 seconds and 72°C for 45 seconds, followed by 72°C for 10 minutes. Quality and intensity of the product amplified were verified in 1% agarose gel electrophoresis.

**MicroRNA-target transcripts RT-qPCR analysis**

The efficiency of the amplification reaction for each primer pairs was determined by cDNA dilution curve at serial concentrations (60ng, 15ng, 3.8ng, 0.9ng and 0.2ng) and with variation of 10% of the ideal. RNA was converted to cDNA using Amp Grade DNAse (Invitrogen), oligo dT primers 12-18 (GE Healthcare Life Sciences, Little Chalfont, St.Giles, UK), random primers (Invitrogen) and Super Script III Reverse Transcriptase (Invitrogen). The reactions were assembled in duplicate by automatic pipetting (QIAgility System, Qiagen) and submitted to standard cycling on the 7900HT Real Time PCR System (Applied Biosystems, CA, USA).

**Viability assay after 5-Aza-dC treatment**

The cell lines (tested and negative for mycoplasma contamination) were seeded into 96-well microplates (approximately 1×10^4^ cells per well in 200μL of complete medium). After about 70% of confluence, the cells were exposed to different concentrations of 5-Aza-dC (0.1μM, 0.5μM, 1μM, 2μM and 3μM) for 24, 48, 72 and 96 hours. The medium was aspirated, and the cells were incubated with 3-(4,5-dimethylthiazolyl-2)-2, 5-diphenyltetrazolium bromide (MTT) solution (0.5 mg/mL) (Sigma Aldrich, Darmstadt, Germany) for 4 hours at 37°C. This mixture was carefully aspirated and 200μL of DMSO (Sigma) was added to each well. After 10 minutes, the plates were read in a spectrophotometer at 550nM. Negative controls (DMSO treatment and “no cell control”) were conducted under identical conditions and used as a parameter of comparison.

**Global demethylation assay in PTC cell lines**

The cell lines (TPC1 and BCPAP) were seeded (1x10^5^) in duplicate in 25cm^3^ flasks. After 24 hours, 1μM and 3μM of 5-Aza-dC were added to the appropriate culture medium followed by incubation at 37°C for 96 hours. The culture medium was changed to fresh medium containing the same concentration of 5-Aza-dC every 24 hours. The control treatment was performed with DMSO (Sigma-Aldrich, St Louis, Missouri, USA). After the treatment period, the cells underwent a 24-hour recovery period prior to DNA and RNA extraction using the ReliaPrep™ gDNA Tissue Miniprep System kits (Promega, Madison, Wisconsin, USA) and miRNeasy Mini Kit (Qiagen, Valencia, CA), respectively, according to the manufacturer's specifications. The DNA was converted by sodium bisulfite using the EZ DNA Methylation Gold Kit (Zymo, Irvine, CA, USA).

**Supplementary Methods References**

Chen YA, Lemire M, Choufani S, Butcher DT, Grafodatskaya D, Zanke BW *et al*. Discovery of cross-reactive probes and polymorphic CpGs in the Illumina Infinium HumanMethylation450 microarray. *Epigenetic*s. 2013; **8**(2):203-9.

Leek JT, Johnson WE, Parker HS, Jaffe AE, Storey JD. The sva package for removing batch effects and other unwanted variation in high-throughput experiments. *Bioinformatics* 2012; **28**(6):882-883.

Pidsley R, Y Wong CC, Volta M, Lunnon K, Mill J, Schalkwyk LC. A data-driven approach to preprocessing Illumina 450K methylation array data. *BMC Genomics*. 2013; **14**:293.
